# Stool microbiota composition is associated with the prospective risk of *Plasmodium falciparum* infection

**DOI:** 10.1186/s12864-015-1819-3

**Published:** 2015-08-22

**Authors:** Shibu Yooseph, Ewen F. Kirkness, Tuan M. Tran, Derek M. Harkins, Marcus B. Jones, Manolito G. Torralba, Elise O’Connell, Thomas B. Nutman, Safiatou Doumbo, Ogobara K. Doumbo, Boubacar Traore, Peter D. Crompton, Karen E. Nelson

**Affiliations:** J. Craig Venter Institute, 4120 Capricorn Lane, La Jolla, CA 92037 USA; J. Craig Venter Institute, 9704 Medical Center Drive, Rockville, MD 20850 USA; Laboratory of Immunogenetics, Division of Intramural Research, National Institute of Allergy and Infectious Diseases, National Institutes of Health, Rockville, MD 20852 USA; Laboratory of Parasitic Diseases, Division of Intramural Research, National Institute of Allergy and Infectious Diseases, National Institutes of Health, Bethesda, MD 20892 USA; Malaria Research and Training Centre, Department of Epidemiology of Parasitic Diseases, International Center of Excellence in Research, University of Sciences, Technique and Technology of Bamako, Bamako, Mali

**Keywords:** Stool microbiota, Gut microbiota, Malaria, 16S rRNA gene sequencing, Plasmodium falciparum, Human, Prospective cohort

## Abstract

**Background:**

In humans it is unknown if the composition of the gut microbiota alters the risk of *Plasmodium falciparum* infection or the risk of developing febrile malaria once *P. falciparum* infection is established. Here we collected stool samples from a cohort composed of 195 Malian children and adults just prior to an intense *P. falciparum* transmission season. We assayed these samples using massively parallel sequencing of the 16S ribosomal RNA gene to identify the composition of the gut bacterial communities in these individuals. During the ensuing 6-month *P. falciparum* transmission season we examined the relationship between the stool microbiota composition of individuals in this cohort and their prospective risk of both *P. falciparum* infection and febrile malaria.

**Results:**

Consistent with prior studies, stool microbial diversity in the present cohort increased with age, although the overall microbiota profile was distinct from cohorts in other regions of Africa, Asia and North America. Age-adjusted Cox regression analysis revealed a significant association between microbiota composition and the prospective risk of *P. falciparum* infection; however, no relationship was observed between microbiota composition and the risk of developing febrile malaria once *P. falciparum* infection was established.

**Conclusions:**

These findings underscore the diversity of gut microbiota across geographic regions, and suggest that strategic modulation of gut microbiota composition could decrease the risk of *P. falciparum* infection in malaria-endemic areas, potentially as an adjunct to partially effective malaria vaccines.

**Electronic supplementary material:**

The online version of this article (doi:10.1186/s12864-015-1819-3) contains supplementary material, which is available to authorized users.

## Background

The human microbiome represents the trillions of microbes that naturally inhabit the human body, the vast majority of which reside within the gastrointestinal tract [1]. Major advances in sequencing technology have allowed for investigations of the microbiome in the context of health and disease. Large-scale studies of the westernized human microbiome, including the NIH funded Roadmap Human Microbiome Project (HMP) [2, 3] targeting several body sites and the European Commission funded Metagenomics of the Human Intestinal Tract [4], have defined the healthy human microbiome, providing a reference for subsequent studies into the interactions between microbes and their environment in the context of host immunity and/or genetics.

Systematic studies aimed at understanding the complex interactions between the host and microbiota have demonstrated that the composition of the gut microbiota varies with age [5–7], race/ethnicity [2], geographical location [5, 6], and dietary intake [7–9]. The role of the microbiota in modulating host immune responses to infectious pathogens is also increasingly recognized [10]. Indeed, the composition of the gut microbiota has been shown to influence both local [11–13] and systemic [14–16] immune responses.

Among parasitic pathogens, *Plasmodium falciparum* malaria, which is transmitted by *Anopheles* mosquitos, imposes the greatest disease burden worldwide. The World Health Organization (WHO) estimates that 3.3 billion people are at risk of malaria in 97 countries. In 2013, there were approximately 198 million cases of malaria and an estimated 584,000 deaths, 90 % of which occurred in Africa, with children under 5 years accounting for 78 % of all deaths [17].

In areas of intense malaria transmission, immunity to life-threatening malaria is typically acquired by early childhood, whereas older children remain susceptible to repeated bouts of febrile malaria through adolescence, eventually acquiring near-complete immunity to the disease caused by blood-stage malaria parasites by early adulthood [18, 19]. However, despite repeated infections over years, sterilizing acquired immunity to *P. falciparum* infection seems to occur rarely [20]. The nature of the human immune response that protects from *P. falciparum* infection and the disease it causes is complex and only partially understood [21]. Indeed, the quality of the immune response and the clinical outcome during any given *P. falciparum* infection can vary greatly between and within individuals [22]. Although host and parasite genetic factors [23, 24] clearly contribute to the heterogeneity in immune and clinical responses to malaria, it is likely that environmental factors such as the host microbiota also play a role. For example, a recent mouse study demonstrated that anti-α-gal antibodies—induced by the presence of *Escherichia coli* O86:B7 in the gut—are cytotoxic to α-gal-expressing *Plasmodium* sporozoites, and thus protect mice from mosquito-transmitted *Plasmodium* infection [25]. In the same report, an association was observed between anti-α gal IgM levels and protection from *P. falciparum* infection in Malian children and adults residing in an area of intense malaria transmission [25], suggesting that the host microbiota may play a role in susceptibility to *P. falciparum* infection in humans. In addition, Li *et al.* [26] noted that experimental infection of mice with *Plasmodium berghei* resulted in altered metabolite profiles that they attributed to a disturbance of the gut microbial community caused by the parasite. However, it is currently unknown in humans if the composition of the gut microbiota modulates the risk of *P. falciparum* infection or the risk of developing malarial disease once *P. falciparum* blood-stage infection is established.

To investigate the potential interplay between *P. falciparum* and the gut microbiota, we analyzed the bacterial communities in stool samples collected from a cohort of 195 Malian children and adults just prior to an intense malaria transmission season. Community composition was determined by sequencing the 16S ribosomal RNA (rRNA) gene using high-throughput next generation sequencing technology. In a prospective cohort study we examined the relationship between the gut microbiota composition of these individuals and their subsequent risk of *P. falciparum* infection and febrile malaria. We also compared the microbiota composition of this cohort with published studies conducted in other regions. Consistent with prior studies, we found that gut microbial diversity in this cohort increased with age, although the overall microbiota profile was distinct from cohorts in other regions of Africa, Asia and North America. Age-adjusted Cox regression analysis revealed a significant association between the microbiota composition before the malaria season and the prospective risk of *P. falciparum* infection during the ensuing malaria season, while no relationship was observed between microbiota composition and the risk of developing febrile malaria once *P. falciparum* infection was established.

## Results

### Characteristics of study subjects

**Fig. 1:**
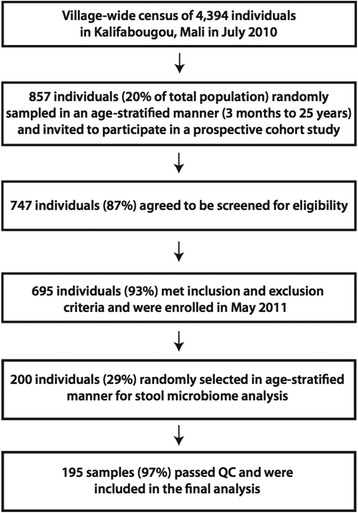
Selection of study subjects for stool microbiota analysis

From May 11 – May 31, 2011 (end of 6-month dry season) we enrolled 695 healthy individuals in this cohort study. Stool was collected from all subjects at the time of enrollment. A subset of 200 individuals was randomly selected in an age-stratified manner for stool microbiota analysis (Fig. 1). Of these 200 subjects, 55 subjects (28 %) were asymptomatically infected with *P. falciparum* blood-stage parasites by PCR at enrollment when stool was also collected. The prevalence of intestinal helminths at enrollment was near zero, consistent with the mass-drug treatment program in this region of Mali, whereas the prevalence of *Schistosoma haematobium* in the urine was 7.4 %, mostly among adolescents and adults. The prevalence of sickle cell trait (HbAS) was 8.5 %. Detailed demographic and clinical data stratified by age are shown in Table 1. Consistent with our findings in the entire cohort [20], longitudinal analysis during the ensuing malaria season showed that the risk of febrile malaria decreased with increasing age in the subset of individuals in this study, with the exception of infants under the age of 1 year whose risk was intermediate between children and adults (Table 2), presumably due to the protective effect of maternal antibodies [27].

**Table 1:** Baseline characteristics of study subjects by age group

Characteristic	Age Group						All (*n* = 200)
	3–5 mo (*n* = 26)	6 mo–1 y (*n* = 41)	2–5 y (*n* = 37)	6–11 y (*n* = 44)	12–17 y (*n* = 24)	18–25 y (*n* = 28)	
Female gender	11 (42 %)	26 (63 %)	16 (43 %)	23 (52 %)	11 (46 %)	19 (68 %)	106 (53 %)
Bambara ethnicity	21 (81 %)	37 (90 %)	32 (86 %)	38 (86 %)	22 (92 %)	24 (86 %)	174 (87 %)
*P. falciparum* PCR positive at enrollment^a^	0 (0 %)	10 (24 %)	11 (30 %)	4 (9.1 %)	16 (67 %)	14 (50 %)	55 (28 %)
*P. malariae* PCR positive at enrollment	0	0	0	0	1 (4.2 %)	0	1 (0.5 %)
Stool PCR for intestinal helminths (number positive/number tested)							
*A. duodenale*				0/15	0/24	0/28	0/67
*A. lumbricoides*				0/11	0/24	0/28	0/63
*N. americanus*				0/22	0/24	1/28 (3.6 %)	1/74 (1.4 %)
*S. stercoralis*				0/11	0/24	0/28	0/63
*T. trichura*				0/11	0/24	0/28	0/63
Kato-Katz for intestinal and urinary helminths (number positive/number tested)							
*S. mansoni* (stool)	0/24	0/40	0/37	0/44	0/24	0/28	0/197
*H. nana* (stool)	0/24	0/40	0/37	0/44	0/24	0/28	0/197
*S.haematobium* (urine)	2/19 (11 %)	1/39 (2.6 %)	0/37 (0 %)	1/43 (2.3 %)	2/16 (12 %)	7/22 (32 %)	13/176 (7.4 %)
Hb type							
AA	23 (88 %)	35 (85 %)	29 (78 %)	38 (86 %)	22 (92 %)	19 (68 %)	166 (83 %)
AC	1 (3.8 %)	3 (7.3 %)	6 (16 %)	2 (4.5 %)	1 (4.2 %)	3 (11 %)	16 (8 %)
AS	2 (7.7 %)	3 (7.3 %)	2 (5.4 %)	4 (9.1 %)	1 (4.2 %)	5 (18 %)	17 (8.5 %)
SC	0	0	0	0	0	1 (3.6 %)	1 (0.5 %)
Hemoglobin, g/dL (95 % CI)	11.0 (10.7 – 11.3)	10.2 (9.9 – 10.6)	11.1 (10.8 – 11.5)	12.3 (12.0 – 12.6)	13.0 (12.6 – 13.4)	13.5 (12.9 – 14.0)	11.7 (11.5 – 12.0)

^a^Before the malaria season and at time of stool collection. All *P. falciparum*-infected subjects at the time of enrollment were asymptomatic

**Table 2:** Malaria outcomes by age group

Outcome	Age Group						P value
	3–5 mo (*n* = 26)	6 mo–1 y (*n* = 41)	2–5 y (*n* = 37)	6–11 y (*n* = 44)	12–17 y (*n* = 24)	18–25 y (*n* = 28)	
Median days from enrollment to first malaria episode^a^ (95 % CI)	227 (159–249)	NE	134 (109 – 176)	142 (120 – 257)	NE	NE	<0.0001*
Median parasite density for first malaria episodes in parasites/μL (interquartile range)	14,400 (9,600 – 47,850)	38,740 (18,210 – 173,400)	19,800 (8,700 – 57,680)	16,300 (5,700 – 76,300)	13,920 (7,412 – 24,000)	10,720 (9,012 – 14,080)	0.19**
Individuals with ≥1 malaria episodes (% of age group)	4 (15 %)	5 (12 %)	13 (35 %)	13 (30 %)	1 (4.2 %)	0	<0.001***

Abbreviations: *CI*, confidence interval; *NE*, not estimated because >50 % of individuals remained free of malaria during the study period

^a^Malaria defined as ≥2500 parasites/μL, an axillary temperature of ≥37.5 °C within 24 h, and no other cause of fever discernible by physical exam

*log-rank test

**Kruskal-Wallis test

*** Fisher Exact Test

### Diversity and composition of the microbiota in Mali stool samples

Sequence data were successfully generated from 195 out of the 200 stool samples. After processing the raw sequence data, the combined dataset consisted of 1,331,167 sequences. However, not all sequences could be confidently assigned taxonomy to the genus level; therefore we denote these sequences by appending the tag *unclassified* to the end of their taxonomic assignment (one of phylum, class, order, or family levels). The taxonomic assignments were used to generate a sample-taxa count matrix. In the combined dataset, *Bifidobacterium*, *Streptococcus*, and family *Ruminococcaceae* unclassified were the three most abundant taxonomic groups, accounting for 13.7 %, 12.9 %, and 12.4 % of sequences, respectively.

**Fig. 2:**
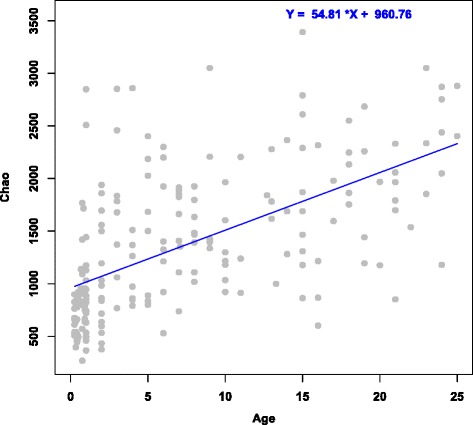
Increase in microbial taxa diversity with age in the Malian cohort. For each stool microbiota sample, the alpha diversity (Chao estimate) of the microbial community (y-axis) was plotted against the individual’s age in years (x-axis). The blue line is the linear model fit and has a positive slope (*P* < 10^−15^)

Operational Taxonomic Units (OTUs) were identified in these data by clustering sequences at 97 % sequence identity, and were used to compute microbial community diversity (alpha diversity) in these samples. Subsequently, the relationship between diversity and age was assessed using a linear model. This analysis revealed that diversity increased with age in this cohort (Fig. 2); a similar trend was observed for both genders separately (Additional file 1: Figure S1).

**Fig. 3:**
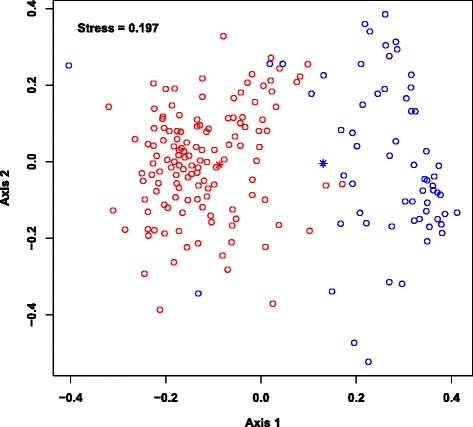
An NMDS ordination of the microbiota samples. The taxonomic profiles of the samples were used to compute a sample dissimilarity matrix (using Bray-Curtis measure) and this matrix was used to compute an ordination of the samples in two dimensions (Axis 1 and Axis 2). The goodness of fit (or stress) associated with this ordination is 0.197. The circles in this plot denote the samples. The two sample groups (or Dirichlet components) identified using the mixture modeling are denoted in red (DC1) and blue (DC2). The average ages for the groups are 10.3 years (DC1) and 1.3 years (DC2). Asterisks denote the vectors of mean taxa proportions associated with the corresponding Dirichlet components

**Fig. 4:**
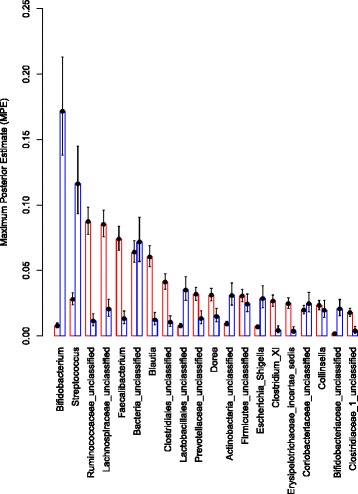
Mean proportions (calculated using Maximum Posterior Estimate) of the twenty most abundant taxa (based on abundance in one of the groups) in the two Dirichlet components: the older DC1 group (*red*) and younger DC2 group (*blue*). The lower and upper 95 % credible intervals are shown as error bars

The sample-taxa count matrix was further analyzed using a Dirichlet Multinomial Mixture (DMM) modeling framework [28] to identify sample groups (or Dirichlet components). An evaluation of the model fit (Additional file 1: Figure S2) revealed that two Dirichlet components (hereafter referred to as DC1 and DC2) provided the best fit for these data. Figure 3 shows a two-dimensional ordination of these samples that was generated using non-metric multidimensional scaling (NMDS) based on Bray-Curtis dissimilarity between samples. The individuals comprising the sample groups DC1 (*n* = 133) and DC2 (*n* = 62) were significantly different in terms of age (*P* < 10^−15^; assessed using a *t*-test) and taxonomic profiles (*P* ≤ 0.05; assessed by non-overlapping 95 % credible intervals), but had similar gender distributions. Specifically, the average ages of individuals in DC1 and DC2 were 10.3 years and 1.3 years, respectively. The most abundant taxa in the older DC1 group were family *Ruminococcaceae* unclassified (8.7 %), family *Lachnospiraceae* unclassified (8.5 %), and *Faecalibacterium* (7.4 %), while the most abundant taxa in the younger DC2 group were *Bifidobacterium* (17.1 %) and *Streptococcus* (11.6 %) (Fig. 4 and Additional file 1: Table S1).

### Comparison of Mali, Malawi, HMP, and MSD cohorts

Next, we compared the gut microbiota composition of the Mali cohort with data from the Malawi, HMP, and MSD microbiome studies described in *Material and Methods*. The four datasets were analyzed based on taxonomies of their constituent samples. DMM modeling identified 9 Dirichlet components (CC1 – CC9) (Additional file 1: Table S2), each with a different taxonomic profile (Additional file 1: Table S3). The 9 Dirichlet sample groups largely fell along cohort lines (Additional file 1: Table S4). For example, the Dirichlet components DC1 and DC2 that were identified by DMM modeling of the Mali cohort were largely preserved as components CC2 and CC5, respectively. Like CC5, which was comprised exclusively of Mali samples, CC6 had a high abundance of *Bifidobacterium* and *Streptococcus*, but only contained samples from the Malawi cohort; however, the proportions of *Bifidobacterium* and *Streptococcus* were different in CC6 (22 % and 5 %, respectively) compared to CC5 (15 % and 11 %, respectively). The HMP samples were predominantly split across CC4 and CC9, both of which were dominated by *Bacteroides*, but at different abundance levels (25 % and 60 %, respectively). The MSD cohort was mostly split across CC1, CC3, and CC8, all of which were dominated by *Prevotella* to varying degrees (8 % in CC1 and 70 % in CC3) and family *Enterobacteriaceae* unclassified (9.6 % in CC8). CC7 consisted of Malawi samples and was also dominated by *Prevotella* (21 %).

### Relationship between gut microbiota and persistent *P. falciparum* infection, incident *P. falciparum* infection, and incident febrile malaria in the Malian cohort

**Fig. 5:**
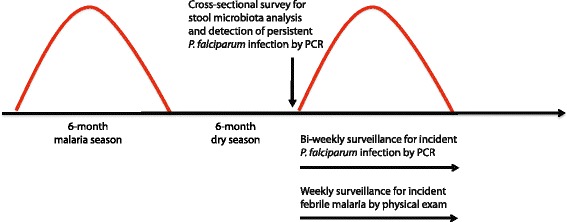
Definition of persistent *P. falciparum* infection, incident *P. falciparum* infection, and incident febrile malaria. This longitudinal cohort study in Mali was designed to take advantage of the sharply demarcated 6-month malaria season (July – December) and 6-month dry season (January – June) during which there is either intense or negligible *P. falciparum* transmission, respectively. Stool microbiota composition was determined for all study subjects in a cross-sectional survey at the end of the 6-month dry season. For each subject, we examined the relationship between stool microbiota composition and the risk of persistent *P. falciparum* infection, incident *P. falciparum* infection and incident febrile malaria. Individuals with *P. falciparum* infections that persisted without symptoms through the dry season were identified by PCR analysis of fingerprick blood samples in a cross-sectional survey at the end of the 6-month dry season (at the time of stool collection). For subjects who began the malaria season without *P. falciparum* infection, incident *P. falciparum* infections during the ensuing malaria season were detected prospectively through bi-weekly PCR analysis of fingerprick blood samples. For all subjects who became infected with P. falciparum blood-stage parasites, incident cases of febrile malaria (axillary temperature ≥37.5 °C and ≥2500 asexual parasites/μl of blood) were detected prospectively during the ensuing malaria season through weekly physical examination by the study physician and blood smear microscopy if malaria was suspected

We investigated the relationship between the gut microbiota composition and three distinct clinical phenotypes in the Malian cohort: persistent *P. falciparum* infection, incident *P. falciparum* infection, and incident febrile malaria. These three phenotypes are described in Fig. 5.

**Fig. 6:**
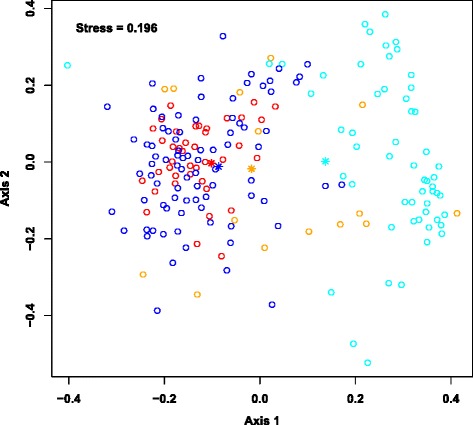
NMDS ordination of the Mali samples on the basis of *P. falciparum* infection status at the end of the 6-month dry season. Samples from subjects with persistent, asymptomatic *P. falciparum* infection at the end of the dry season fall into two components PP1 (*orange*) and PP2 (*red*); samples from *P. falciparum*-uninfected subjects fall into two components as well – PN1 (*cyan*) and PN2 (*blue*). The mean vector of each component is labeled with an asterisk in their respective color. The average ages for the groups are 3.2 years (PP1), 13.6 years (PP2), 1.3 years (PN1) and 9.2 years (PN2)

**Fig. 7:**
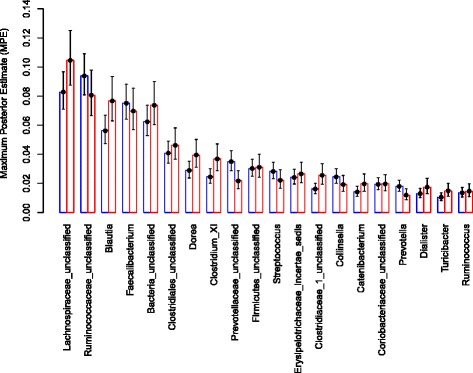
Mean proportions (Maximum Posterior Estimate) of the twenty most abundant taxa in the two Dirichlet components PP2 (*red*) and PN2 (*blue*). The lower and upper 95 % credible intervals are shown as error bars

In a cross-sectional analysis at the end of the 6-month dry season, a period of negligible malaria transmission [29], we first compared the gut microbiota composition of individuals with persistent, asymptomatic *P. falciparum* blood-stage infection versus uninfected individuals. *P. falciparum* infections detected at the end of the 6-month dry season are generally acquired during the preceding transmission season and persist through the dry season at low levels without causing symptoms. This analysis was carried out using the DMM framework, but in a supervised manner with subjects classified as either persistently infected (*n* = 55) or uninfected (*n* = 140). An NMDS ordination of the samples along with their *P. falciparum* infection status at the end of the dry season is shown in Fig. 6. Among the 55 persistently infected subjects we identified two Dirichlet components PP1 (15 subjects) and PP2 (40 subjects), with average subject ages of 3.2 years and 13.6 years, respectively (Additional file 1: Table S5). Among the 140 uninfected subjects we identified two components PN1 (56 subjects) and PN2 (84 subjects) with average subject ages of 1.3 years and 9.2 years, respectively (Additional file 1: Table S5). The mean vector of taxa proportions for component PP2 (older and infected) is very close to that of component PN2 (older and uninfected) indicating that these two subject groups have very similar taxonomic proportions, with no statistically significant differences (Fig. 7, Additional file 1: Table S6). The mean of component PP1 (young and infected) was not as close to the means of either of PN1 or PN2. However, the only taxa in this component that had a significantly different proportion compared to its values in PN1 and PN2 was *Bifidobacterium* (3.6 %). Together, these findings suggest that age may be a stronger predictor of gut microbiota composition than *P. falciparum* infection status at the end of the 6-month dry season (i.e. persistent *P. falciparum* infection versus no infection).

**Fig. 8:**
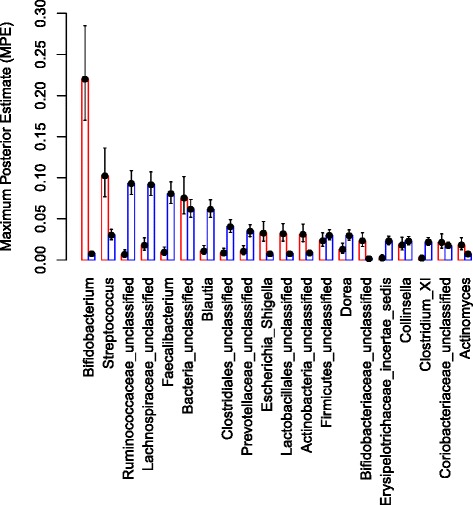
Mean proportions (Maximum Posterior Estimate) of the twenty most abundant taxa in the two Dirichlet components FM1 (*red*) and FM2 (*blue*). The lower and upper 95 % credible intervals are shown as error bars

**Fig. 9:**
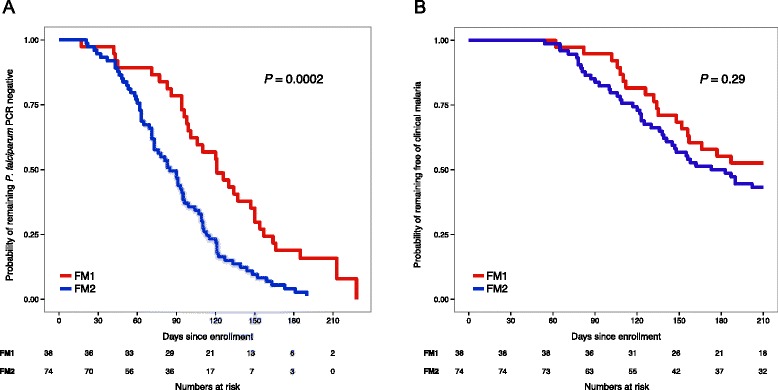
Stool microbiota composition is associated with the prospective risk of *P. falciparum* infection but not the prospective risk of febrile malaria. Kaplan-Meier plots showing time to (**a**) the first PCR-confirmed *P. falciparum* blood-stage infection (irrespective of whether symptoms were present) and (**b**) the first febrile malaria episode among subjects with documented *P. falciparum* infection, stratified by the two DMM sample groups FM1 and FM2. *P* values comparing FM1 to FM2 were calculated by the log-rank test

Next, we examined the relationship between the stool microbiota composition present before the malaria season and the prospective risk of becoming infected with *P. falciparum* blood-stage parasites during the ensuing 6-month malaria season. For this analysis we only included subjects who were not infected with *P. falciparum* (by PCR) before the malaria season and who had completed study follow-up through the end of the ensuing 6-month malaria season (112 subjects). DMM modeling of the microbiota data obtained from these *P. falciparum* uninfected subjects identified two Dirichlet components FM1 (38 subjects) and FM2 (74 subjects). These two sample groups had distinct taxonomic profiles (Additional file 1: Table S7, Fig. 8). *Bifidobacterium* (22 %) and *Streptococcus* (10 %) were the most abundant taxa in FM1 while family *Ruminococcaceae* unclassified (9.3 %), and family *Lachnospiraceae* unclassified (9.1 %) were the most abundant taxa in FM2. In addition to these taxa, other taxa including *Faecalibacterium*, *Blautia*, order *Clostridiales* unclassified, family *Prevotellaceae* unclassified, and *Escherichia/Shigella* were differentially abundant across the two sample groups (P ≤ 0.05; assessed by non-overlapping 95 % credible intervals). The average age of subjects in FM1 and FM2 was 1.4 years and 9.1 years, respectively. During the ensuing malaria season, incident (new) *P. falciparum* blood-stage infections in these subjects were detected through bi-weekly PCR analysis of fingerprick blood samples. We compared the time to first *P. falciparum* infection in the FM1 versus FM2 sample groups (irrespective of whether the infection caused symptoms) by generating Kaplan-Meier plots and performing log-rank analysis (Fig. 9). We noted a statistically significant delay in time to first *P. falciparum* infection (Fig. 9a) for FM1 subjects (median: 121 days; 95 % CI 101–150) compared to FM2 subjects (median: 85 days; 95 % CI 73–99; P = 0.0002). Moreover, Cox regression analysis accounting for age, gender, anemia, HbAS, *S. haematobium* infection, splenomegaly and distance to the river (Additional file 1: Table S8) revealed a statistically significant increased risk of *P. falciparum* infection for FM2 subjects versus FM1 subjects (hazards ratio = 2.41; 95 % CI: 1.33-4.36, *P* = 0.0038).

Finally, we examined the relationship between the stool microbiota composition present before the malaria season and the prospective risk of developing febrile malaria in subjects who became infected with *P. falciparum* blood-stage parasites during the ensuing malaria season. Again, for this analysis we only included subjects who were PCR negative for *P. falciparum* infection before the malaria season and then became infected with *P. falciparum* during the ensuing malaria season, as detected through bi-weekly PCR of fingerprick blood samples. Malaria immune subjects (*n* = 49) were defined as those whose *P. falciparum* infections never progressed to febrile, symptomatic malaria despite weekly active clinical surveillance; whereas malaria susceptible subjects (*n* = 63) were defined as those whose *P. falciparum* infection progressed to a febrile malaria episode (T ≥ 37.5 °C and parasite density ≥ 2500 asexual parasites/μl). On the basis of the DMM modeling of the microbiota data described above, the same FM1 and FM2 sample groups had similar proportions of malaria immune and susceptible subjects (FM1: 18 immune and 20 susceptible; FM2: 31 immune and 43 susceptible; P = 0.55), suggesting that gut microbiota composition is not associated with the risk of febrile malaria once a *P. falciparum* blood-stage infection is established. On the basis of the same Dirichlet sample groups FM1 and FM2, we also generated Kaplan-Meier plots and performed log-rank analysis for time to first febrile malaria episode (Fig. 9b). We found no statistically significant difference between FM1 and FM2 for time to first febrile malaria episode using either the case definition for clinical malaria (T ≥ 37.5 °C and parasite density ≥2500 asexual parasites/μl) or the less restrictive definition of fever and any level of *P. falciparum* parasitemia.

## Discussion

In this study we aimed to describe the composition of the gut microbiota in an age-stratified rural West African population that is exposed to intense seasonal *P. falciparum* transmission. We compared the gut microbiota composition in this population with studies conducted in other regions. In an exploratory analysis we also investigated the relationship between the gut microbiota composition and the prospective risk of *P. falciparum* infection as well as the risk of developing febrile malaria once *P. falciparum* infection is established.

Like studies conducted in other regions (for instance, [5]) we found that the alpha diversity of the gut microbiota increased with age in both males and females. We found in this cohort that the most dynamic shifts in microbiota composition occur before the 3–5 years of age, possibly related to the transition from breast milk to solid foods [30] and then changes occur more gradually through adolescence and adulthood. We observed clear differences between the composition of the gut microbiota in this study and that reported in studies conducted in other parts of the world, underscoring the diversity of gut microbiotas across geographic regions. Although the non-uniformity of the 16S variable region that was selected for sequencing across these studies may have impacted taxa detection [31], several sample groups shared similar taxa, albeit at different abundance levels. The relative impact of factors underlying the observed variability in microbiota composition across geographic regions such as diet, antibiotic exposure, genetics, and co-infections remains to be determined.

Using a DMM framework we identified two subject groups with distinct microbiota profiles (FM1 and FM2) that exhibited statistically significant differences in the prospective risk of *P. falciparum* infection after accounting for age and other potential confounders. The gut microbiota of subjects at lower risk of *P. falciparum* infection (FM1) contained a significantly higher proportion of *Bifidobacterium* and *Streptococcus* compared to subjects at higher risk of *P. falciparum* infection (FM2). This finding raises the possibility that modulation of the microbiota with pre- and/or pro-biotic nutritional supplementation that promotes the growth of commensal organisms like *Bifidobacterium,* particularly in the neonatal period [32, 33], may be of benefit in the context of malaria. Importantly, studies indicate that natural components of human milk stimulate the growth of *Bifidobacterium* [34–36]. Clearly, further studies are needed to understand the potential interactions between specific components of the microbiota, innate/adaptive immune responses, and susceptibility to malaria and other pathogens that commonly affect this population.

Also of potential interest, the gut microbiota of FM1 subjects contained a significantly higher proportion of the *Enterobacteriaceae Escherichia/Shigella* compared to subjects at higher risk of *P. falciparum* infection (FM2) (3.2 % versus 0.7 %, respectively) - an intriguing finding in light of a recent mouse study in which anti-α-gal antibodies induced by the presence of α-gal-expressing *Escherichia coli* in the gut, were cytotoxic to α-gal-expressing *Plasmodium* sporozoites in the skin, thus protecting mice from *Plasmodium* infection [25]. Because not all *Enterobacteriaceae* express α-gal [37] we are unable to ascertain from the present 16S data whether strains in these samples express α-gal. A functional screen for the expression of this gene or a metagenomic approach would be required to answer this question. It will be of interest in future studies to use higher resolution techniques to determine if differences in the proportion of α-gal expressing *Enterobacteriaceae* are associated with the risk of *P. falciparum* infection. Moreover, additional studies are needed in various malaria-endemic areas to determine if the findings here can be replicated, since the association we observed between microbiota composition and *P. falciparum* infection risk may have occurred by chance or may be confounded by other factors not measured in this study.

Importantly, we did not observe a relationship between the gut microbiota composition and the prospective risk of developing febrile malaria once *P. falciparum* blood-stage infections were established, possibly due to a lack of statistical power. However, we observed clear changes in the microbiota composition over the age range during which clinical immunity to febrile malaria is slowly acquired in areas of intense malaria transmission [20], suggesting that age-associated changes in the gut microbiota from infancy to adulthood may play a role in shaping the quality of the immune response to malaria as individuals gradually acquire clinical immunity to malaria, hypotheses that could be further tested in larger longitudinal studies.

## Conclusions

The findings of this study underscore the diversity of the gut microbiota with age and across geographic regions. In addition, the preliminary finding of an association between gut microbiota composition and *P. falciparum* infection risk suggests that strategic modulation of gut microbiota composition could decrease *P. falciparum* infection risk in malaria-endemic areas, potentially as an adjunct to partially effective malaria vaccines.

## Methods

### Ethics statement

The Ethics Committee of the Faculty of Medicine, Pharmacy and Dentistry at the University of Sciences, Technique and Technology of Bamako, the Institutional Review Board (IRB) of the National Institute of Allergy and Infectious Diseases (NIAID), National Institutes of Health (NIH) and of the J. Craig Venter Institute (JCVI) approved this study. Written, informed consent was obtained from adult subjects and from the parents or guardians of participating children.

### Study site and subjects

This cohort study was conducted in Mali in the rural village of Kalifabougou which has a population of 4,394 and is situated ~40 km northwest of the capital Bamako. Bambara is the predominant ethnic group and ~90 % of residents engage in subsistence farming. Kalifabougou is in the savanna ecoclimatic zone where annual rainfall is 800–1,200 mm/year. Intense transmission of *P. falciparum* malaria occurs during the rainy season in this region of Mali from June through December. In May 2011, we enrolled 695 healthy individuals aged 3 months to 25 years in a cohort study to investigate mechanisms of naturally acquired immunity to malaria using systems biology approaches. A detailed description of the cohort has been published elsewhere [38]. As part of the initial clinical assessment, spleen size was scored using the Hackett method [39], with splenomegaly defined as a Hackett score >1. Baseline hemoglobin values, measured by a HemoCue analyzer, were used to determine anemia status based on WHO criteria, and hemoglobin typing was performed with a D-10 instrument (Bio-Rad) to identify individuals with sickle cell trait (HbAS). Exclusion criteria at enrollment included a hemoglobin level <7 g/dL, axillary temperature ≥37.5 °C, acute systemic illness, underlying chronic disease, use of antimalarial or immunosuppressive medications in the past 30 days, or pregnancy. As part of a mass drug administration program in Mali [40, 41] all residents greater than 5 years of age received albendazole, ivermectin, and praziquantel in March 2011 (prior to enrollment) and only albendazole and ivermectin in October 2011. For the stool microbiota analysis described here, we randomly selected an age-stratified subset of 200 individuals from the entire cohort; the selection process is shown in Fig. 1.

### Surveillance for symptomatic and asymptomatic *Plasmodium* infection

After enrollment, individuals were followed during the ensuing malaria season for 7 months. Symptomatic malaria episodes were detected prospectively by self-referral and through weekly active clinical surveillance. All individuals with signs and symptoms of malaria and any level of *Plasmodium* parasitemia detected by light microscopy were treated according to the National Malaria Control Program guidelines in Mali. Thick blood smears were stained with Giemsa and counted against 300 leukocytes. Parasite densities were recorded as the number of asexual parasites/μl of blood based on a mean leukocyte count of 7500 cells/μl. Each smear was read in a blinded manner by two certified microscopists. The research definition of clinical malaria was parasitemia of ≥2500 parasites/μL, an axillary temperature of ≥37.5 °C within 24 h, and no other cause of fever discernible by physical exam. To detect asymptomatic *Plasmodium* infections during the 7-month study period, blood was collected by fingerprick onto filter paper at bi-weekly scheduled visits. At the end of the study period, stored filter papers were analyzed by PCR chronologically from enrollment onwards until the first *P. falciparum* infection of the malaria season was detected for each individual. A detailed description of the PCR protocol was published elsewhere [20].

### Detection of urinary and intestinal parasites

Stool and urine samples were collected at enrollment. *S. haematobium* eggs were quantified by microscopy after filtration of fresh urine samples with Nytrel filters (Vestergaard Frandsen). *Schistosoma mansoni* and other geohelminth eggs were detected by microscopy of duplicate fresh fecal thick smears using the Kato-Katz method [42]. Aliquots of stool were cryopreserved at −80 °C for DNA extraction and multi-parallel, real-time PCR for intestinal nematodes (*Necator americanus, Ancylostoma duodenale, Trichuris trichiura, Ascaris lumbricoides*, and *Strongyloides stercoralis*) was performed as previously described [43]. Individuals diagnosed with urinary schistosomiasis were treated with praziquantel (see Table 1).

### Stool collection and microbial DNA extraction

The sub-cohort that is the primary focus of this study was comprised of 200 subjects—106 females and 94 males ranging in age from 3 months to 25 years. Stool samples were collected at study enrollment in May 2011 just prior to the six-month malaria season. Stool samples were stored in −80 °C freezers in Mali and shipped to the U.S. on dry ice for analysis. Microbial DNA for 16S rDNA gene sequencing was isolated from stool samples using enzymatic lysis followed by phenol-chloroform isoamyl alcohol extraction and ethanol precipitation. Standard Roche protocols were used for sample preparation.

### 16S rRNA gene sequencing

The 16S rRNA gene amplicons were quantified and pooled in equal mass ratios such that amplicons derived from samples to be analyzed could be multiplexed in a single Roche-454 sequencing run. Established 454 FLX sequencing methods (Roche) that were developed for the HMP, including universal PCR primers with unique barcode identifiers, were used to amplify and sequence the hypervariable regions (V1-V3) of the bacterial 16S rRNA gene [31].

### Comparative dataset analysis

16S rRNA gene sequence data generated from stool samples collected as part of three other studies were included in the analysis component of this study for comparative purposes only. These additional studies included a cohort in Malawi [5], the HMP cohort [44], and the healthy controls from the moderate-to-severe diarrhea (MSD) study [6]. Data for the Malawi cohort was retrieved from MG-RAST (http://metagenomics.anl.gov/). The Malawi cohort was part of accession “mgp401” (http://metagenomics.anl.gov/linkin.cgi?project=401) [5]. This study sequenced the V4 region of the 16S rRNA gene using Illumina HiSeq 2000. We were primarily interested in the Malawi cohort because of similarities in age distribution and geographic location. Specifically, the Malawi cohort was comprised of 114 subjects ranging in age from 1.5 weeks to 44 years (age data for 31 subjects were unavailable) and consisted of 63 females and 20 males (gender data for the remaining subjects were unavailable). In common with the present Mali cohort, sequences from the HMP cohort were derived from the V1-V3 regions of the stool bacterial 16S rRNA gene using 454 pyrosequencing. These samples originated from 140 stool samples collected and processed during the HMP. Raw sequence data and metadata were retrieved from the HMP Data Analysis and Coordination Center (HMPDACC) (http://www.hmpdacc.org/). The subjects in the HMP cohort ranged in age from 18 to 40 years and 73 were females and 67 males. Data for the MSD study were downloaded from SRA at NCBI and were available under the bioproject accession PRJNA234437. In the MSD study sequence data was generated (using 454 FLX sequencing) from the V1-V2 region of the 16S rRNA gene from DNA extracted from stool samples. Healthy control samples in the MSD study were identified from the full data set using the available study meta-data and only these (493 in total) were included in the comparison here. The healthy control samples in the MSD study were collected from individuals residing in Bangladesh (117), The Gambia (134), Kenya (140) and Mali (102). These individuals were all under 5 years of age (ranging from 1 to 58 months) and no gender information was available from the study metadata.

### 16S rRNA sequence data processing

For the 454 data, the raw flowgram files (Standard Flowgram Format) were deconvolved using the sample barcode information, and the barcodes and primers were trimmed subsequently. All processing of the 454 and Illumina sequences from the various studies were carried out using mothur v1.28 [45] - quality check, sequence screening and filtering, chimera checking, and taxonomy assignment steps used the HMP SOP for 16S rRNA gene (16S) curation (http://www.hmpdacc.org/HM16STR/). OTUs at 97 % sequence identity were computed for the Malian cohort in order to assess sub-genus diversity and structure. The comparisons of the present Malian cohort to the other three datasets were carried out only at the genus level.

For the Malian cohort, from the initial 200 samples, 195 samples were successfully sequenced and each sample yielded more than 1,000 16S rRNA sequences after barcode and primer trimming. Together, the samples resulted in 1,500,243 sequences that were used for downstream analysis. Reads were aligned against a reference template, trimmed (keeping only > =100 bp sequences), and checked for chimeras. The resulting output consisted of 1,331,167 sequences. These were assigned taxonomy down to the genus level using mothur's implementation of the RDP classifier (using a bootstrap confidence threshold of 80 %). For OTU analysis, this sequence set was processed further by alignment column trimming, in which the required length of trimmed sequences was > =200 bp. This yielded 968,362 total sequences. These were clustered at 97 % sequence identity to generate OTUs.

### Statistical analysis of the 16S data

The taxonomic assignments of the 16S sequences were used to generate sample-taxa count matrices. These matrices were analyzed using a DMM modeling framework [28] to identify sample groups, where each sample group (also known as a Dirichlet component or cluster) can be summarized by a vector of taxa proportions. The optimal number of Dirichlet components along with the mean proportion of each taxa in a Dirichlet component was calculated using the software developed by Holmes *et al.* [28]. The program was run for various values of *K,* the number of components, and subsequently, the *K* with the best model fit using the Laplace approximation to the negative log model evidence was chosen as the optimal value for that dataset. The Dirichlet component means were calculated from the Maximum Posterior Estimates (MPE) of the model hyperparameters [28].

### Statistical analysis of the clinical data

For continuous variables we compared the differences between group medians with the Kruskal-Wallis test (Table 2); for binary variables, group comparisons were performed using Fisher’s exact test. Kaplan-Meier curves were used to estimate the probability of remaining free of clinical malaria. For individuals who began the malaria season PCR-negative for *Plasmodium*, Kaplan-Meier curves were also used to estimate the probability of remaining free of blood-stage *P. falciparum* infection. Individuals who were infected with *Plasmodium malariae* were censored at the time of infection. Log-rank analysis was used to test the significance of differences in time to first febrile malaria episode and time to first *P. falciparum* infection between DMM clusters. A Cox proportional hazards model was used to estimate the risk of *P. falciparum* infection between DMM clusters and included the following potentially confounding variables: age (per one year increase), HbAS, gender, mild anemia at enrollment, *S. haematobium* infection, presence of splenomegaly at enrollment (Hackett score >1) and distance from home to river (largest stream in Kalifabougou; per 100 m increase). This combination of variables met the assumptions of proportional hazards. Of these variables, HbAS has been shown to affect the risk of *P. falciparum* infection [46, 47], and we have evidence to suggest that baseline splenomegaly increases subsequent to *P. falciparum* infection risk (unpublished data). Statistical significance was defined as a 2-tailed *P* value of <0.05. We performed all analyses in R version 3.1.1 (http://www.R-project.org).

### Availability of supporting data

The data sets supporting the results of this article are included within the article (and its additional file(s)). The sequence data for the Malian cohort are available at NCBI’s Sequence Read Archive under BioProject PRJNA285808. These data are also available at MG-RAST under Project 4793.

## Additional file

Additional file 1: Table S1. Taxonomic composition of Dirichlet components DC1 and DC2. **Table S2.** Fit of DMM modeling for various values of K (the number of Dirichlet components). Best fit is highlighted in red. **Table S3.** Taxonomic composition of Dirichlet components CC1 through CC9. **Table S4**. Distribution of samples from the four cohorts (Mali, HMP, Malawi, and MSD) in the 9 Dirichlet components (CC1 through CC9). **Table S5.** Fit of DMM modeling for various values of K (the number of Dirichlet components) for *P. falciparum* infection status analysis. Best fit is highlighted in red. **Table S6.** Taxonomic composition of Dirichlet components PP1, PP2, PN1, and PN2. **Table S7.** Taxonomic composition of Dirichlet components FM1 and FM2. **Table S8.** Cox model of DMM Component: Time to Infection. **Figure S1.** Increase in microbial taxa diversity with age in the Malian cohort. For each stool microbiota sample, the alpha diversity on the y-axis was plotted against the individual’s age in years (x-axis). The blue lines are the linear model fits and all of them have positive slope (*P* < 10^−9^). Four different alpha diversity measures are reported (Sobs – the observed #OTUs, Chao estimate, Ace estimate, and Shannon diversity). Similar trends are observed when the datasets are analyzed separately by gender. **Figure S2.** Evaluation of model fit of Dirichlet mixtures to the Mali dataset. Model fit is evaluated using the Laplace approximation to the model evidence for varying values of K (the number of Dirichlet components). For these data, K = 2 results in the best fit. (ZIP 595 kb)

## Abbreviations

HMP: Human Microbiome Project
PCR: Polymerase Chain Reaction
rRNA: ribosomal ribonucleic acid
MSD: Moderate-to-severe diarrhea
DMM: Dirichlet Multinomial Mixture
MPE: Maximum Posterior Estimates
