# Supplementary material for: Stool microbiota composition is associated with the prospective risk of Plasmodium falciparum infection
Source: BMC Genomics. 2015 Aug 22;16(1):631. doi: 10.1186/s12864-015-1819-3 (PMC4546150; doi:10.1186/s12864-015-1819-3)
Supplement: Additional file 1: Table S1. — Taxonomic composition of Dirichlet components DC1 and DC2. Table S2. Fit of DMM modeling for various values of K (the number of Dirichlet components). Best fit is highlighted in red. Table S3. Taxonomic composition of Dirichlet components CC1 through CC9. Table S4. Distribution of samples from the four cohorts (Mali, HMP, Malawi, and MSD) in the 9 Dirichlet components (CC1 through CC9). Table S5. Fit of DMM modeling for various values of K (the number of Dirichlet components) for P. falciparum infection status analysis. Best fit is highlighted in red. Table S6. Taxonomic composition of Dirichlet components PP1, PP2, PN1, and PN2. Table S7. Taxonomic composition of Dirichlet components FM1 and FM2. Table S8. Cox model of DMM Component: Time to Infection. Figure S1. Increase in microbial taxa diversity with age in the Malian cohort. For each stool microbiota sample, the alpha diversity on the y-axis was plotted against the individual’s age in years (x-axis). The blue lines are the linear model fits and all of them have positive slope (P < 10−9). Four different alpha diversity measures are reported (Sobs – the observed #OTUs, Chao estimate, Ace estimate, and Shannon diversity). Similar trends are observed when the datasets are analyzed separately by gender. Figure S2. Evaluation of model fit of Dirichlet mixtures to the Mali dataset. Model fit is evaluated using the Laplace approximation to the model evidence for varying values of K (the number of Dirichlet components). For these data, K = 2 results in the best fit. (ZIP 595 kb) [file 12864_2015_1819_MOESM1_ESM.zip › Supplementary_tables_and_figures/Supplementary_Figures_and_Tables.docx]

**Supplementary Figure 1.** Increase in microbial taxa diversity with age in the Malian cohort. For each stool microbiota sample, the alpha diversity on the y-axis was plotted against the individual’s age in years (x-axis). The blue lines are the linear model fits and all of them have positive slopes (*P* < 10^-9^). Four different alpha diversity measures are reported (Sobs – the observed #OTUs, Chao estimate, Ace estimate, and Shannon diversity). Similar trends are observed when the datasets are analyzed separately by gender.

**Supplementary Figure 2.** Evaluation of model fit of Dirichlet mixtures to the Mali dataset. Model fit is evaluated using the Laplace approximation to the model evidence for varying values of K (the number of Dirichlet components). For these data, K=2 results in the best fit.

**Supplementary Table 1** (attached)

**Supplementary Table 2**. Fit of DMM modeling for various values of K (the number of Dirichlet components). Best fit is highlighted in red.

| **K** | **Model fit using Laplace approximation** |
| --- | --- |
| 1 | 357020.465 |
| 2 | 352396.376 |
| 3 | 347821.089 |
| 4 | 340513.860 |
| 5 | 336412.804 |
| 6 | 333899.776 |
| 7 | 333074.206 |
| 8 | 333046.543 |
| 9 | 333016.536 |
| 10 | 333067.370 |

**Supplementary Table 3** (attached)

**Supplementary Table 4**. Distribution of samples from the four cohorts (Mali, HMP, Malawi, and MSD) in the 9 Dirichlet components (CC1 through CC9).

| **Component number** | **Sample source** | **Number of samples in Component** |
| --- | --- | --- |
| 1 | MSD_Bangladesh | 42 |
| 1 | MSD_Gambia | 31 |
| 1 | MSD_Kenya | 26 |
| 1 | MSD_Mali | 24 |
| 1 | Mali | 4 |
| 1 | HMP | 2 |
| 2 | Mali | 125 |
| 2 | MSD_Bangladesh | 1 |
| 2 | MSD_Kenya | 1 |
| 2 | MSD_Mali | 1 |
| 3 | MSD_Gambia | 67 |
| 3 | MSD_Kenya | 59 |
| 3 | MSD_Mali | 26 |
| 3 | MSD_Bangladesh | 19 |
| 3 | Mali | 1 |
| 4 | HMP | 74 |
| 4 | MSD_Bangladesh | 2 |
| 4 | MSD_Mali | 1 |
| 5 | Mali | 64 |
| 6 | Malawi | 45 |
| 7 | Malawi | 69 |
| 8 | MSD_Kenya | 52 |
| 8 | MSD_Bangladesh | 51 |
| 8 | MSD_Mali | 50 |
| 8 | MSD_Gambia | 35 |
| 8 | Mali | 1 |
| 9 | HMP | 36 |
| 9 | MSD_Bangladesh | 2 |
| 9 | MSD_Kenya | 2 |
| 9 | MSD_Gambia | 1 |

**Supplementary Table 5**. Fit of DMM modeling for various values of K (the number of Dirichlet components) for *P. falciparum* infection status analysis. Best fit is highlighted in red.

| **Number of components K** | **Model fit (persistently infected group)** | **Model fit (uninfected group)** |
| --- | --- | --- |
| 1 | 13772.607 | 30123.139 |
| 2 | 13705.517 | 28941.973 |
| 3 | 13859.548 | 28981.600 |
| 4 | 13982.903 | 29124.867 |

**Supplementary Table 6** (attached)

**Supplementary Table 7** (attached)

**Supplementary Table 8** (attached)
